# Antibiotics Self Medication among Children: A Systematic Review

**DOI:** 10.3390/antibiotics11111583

**Published:** 2022-11-09

**Authors:** Fabrizio Bert, Christian Previti, Francesco Calabrese, Giacomo Scaioli, Roberta Siliquini

**Affiliations:** 1 Department of Public Health and Pediatric Sciences, University of Turin, 10126 Turin, Italy; 2 Hygiene and Infection Control Unit, ASL TO3, 10098 Turin, Italy; 3 AOU City of Health and Science of Turin, 10126 Turin, Italy

**Keywords:** antibiotics, self-medication, children, pre-school, parents, scholar-age

## Abstract

The phenomenon of bacterial antimicrobial resistance (AMR) is a rapidly growing global problem. Overuse and misuse of antibiotics as well as self-prescription are among the most important causes contributing to the growth of antibiotic resistance in humans. This systematic review describes the phenomenon of antibiotics self-medication (ASM) in children. The study was conducted following the Preferred Reporting Items for Systematic Reviews and Meta-Analyses (PRISMA) checklist by searching PubMed, Scopus, and Web of Science until July 2022. Published English language studies containing information regarding parents knowledge, attitudes, and behaviors in self-administration of antibiotics in children were included. A total of 702 articles were identified, and 57 were selected. A higher prevalence of ASM among children was found in the Middle-East (34%), Africa (22%), Asia (20%) and South America (17%), while the lowest prevalence was found in Europe (8%). High distance from hospital, and low income, such as having more than one child, are related with an increased risk of ASM in children. Fever and cough can also promote the misuse of antibiotics by parents. A greater attention to the regulation of the sale of antimicrobial drugs can certainly limit the risk of self-medicating behavior.

## 1. Introduction

The phenomenon of bacterial antimicrobial resistance (AMR), which occurs when changes in bacteria cause drugs used to treat infections to become less effective, is a rapidly growing global problem [1]. In 2017, the World Health Organization first published a document with the 12 families of bacteria that, due to their particular antibiotic resistance mechanisms, are considered dangerous to human health [2].

Specifically, each year worldwide, AMR is directly involved in more than 1.27 million deaths and contributes to another 4.95 million deaths [1]. Because of this enormous impact on human health, in 2019 WHO listed AMR as one of the top ten health threats to the global population [3].

Leading international public health organizations have highlighted the need for early action through prevention strategies and countermeasures to limit the spread of multidrug-resistant microorganisms [4,5]. Among the causes investigated, overuse and misuse of antibiotics are among the most important causes contributing to the growth of antibiotic resistance in humans. In particular, misuse of antimicrobial drugs can occur either through poor adherence to therapy or self-medication [6].

Antibiotic self-medication (AMS) refers to the purchase and use of antibiotic drugs without consulting a physician, as well as storing drugs previously used to treat infections in the home in order to more quickly resolve health problems that are considered similar [7]. Numerous studies have shown that the phenomenon of self-medication with antimicrobial drugs is constantly growing, both in adults and children [8,9]. Often, indeed, having used a drug to treat a previous infection can transmit, in parents, the mistaken belief that the antibiotic can also be used to treat similar symptoms in their children [10].

In children, precisely because of their increased susceptibility, upper airway infections (URTIs) are very common [11]. However, most of these infections are caused by viruses [12]. Thus, the use of antibiotic drugs self-administered by parents can lead to misuse, which can have important implications for children’s health (e.g., side effects, allergies) and, on the other hand, promotes the spread of antibiotic resistance [13].

Numerous studies in the literature show that socio-cultural factors, particularly educational level, socioeconomic status, and nationality, are important in influencing self-medication among adolescents [14,15] and adults [16,17]. To our knowledge, however, there are no systematic reviews examining parental self-medication of children. The purpose of this systematic review is to describe the phenomenon of antibiotic administration in children by parents without a physician’s prescription. In addition, through the analysis of parents’ knowledge, attitudes, and behaviors, we analyze how socio-demographic and geographic variables are involved in the phenomenon of self-medication in children.

## 2. Results

**Figure 1:**
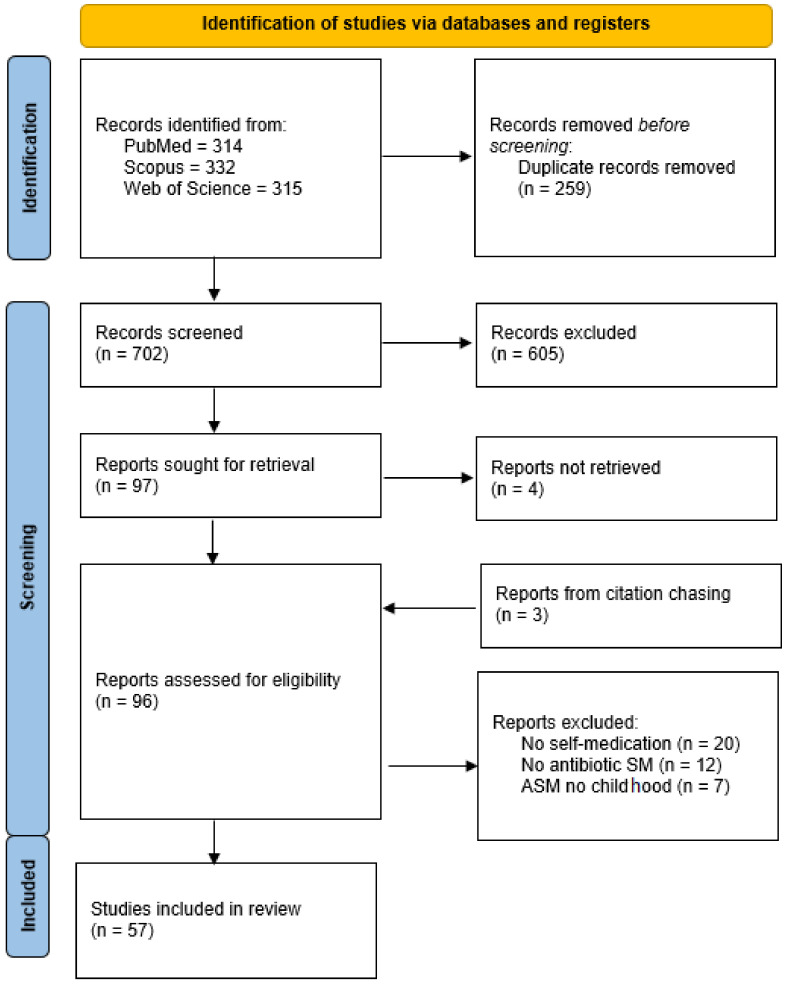
Prisma flow chart.

A total of 961 articles were found in PubMed, Scopus, and Web of Science (314, 332, and 315, respectively). Other three article were identified from citation chasing. After the presence of duplicates was assessed a total of 702 articles were screened following the PRISMA Flow Diagram (Figure 1). An overall of 605 studies were excluded based on non-pertinent title or abstract while the remaining ninety seven were full-text read. Among them 43 were excluded due to various reasons: 20 article did not deal with self-medication, 12 did not deal with antibiotics self-medication, 7 deal with antibiotic self-medication but they did not focus on childhood, and 4 were not available.

### 2.1. Study Characteristics

In total, 57 studies were examined, 56 were cross-sectional articles while one was a systematic review. All studies contained information about antibiotic self-medication attitude. The sample size varied consistently with a minimum number of participants of 85 caregiver and a maximum number of 9526. The average of participants was 1471 and the standard deviation was 2075.

Geographically, the studies analyzed were conducted in Asia (n = 19), the Middle East (n = 11), Africa (n = 9), Europe (n = 9), South America (6), U.S.A. (n = 2), and the Caribbean (n = 1). The studies more frequently came from China (n = 11), Nigeria (n = 5), India (n = 4), Brazil (n = 3), and Saudi Arabia (n = 3).

### 2.2. Antibiotics Self-Medication (ASM)

Prevalence of antibiotics self-medication among children was always obtained from surveys. In an Iraqi study, only parents who self-medicated their children were selected [18].

**Figure 2:**
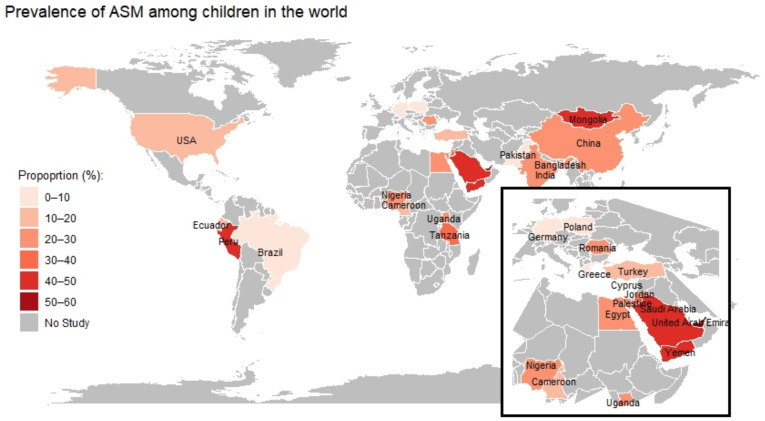
World map showing prevalence of self-medication with antibiotics in children.

On average, parents who have self-medicated their children or who would have were 24%. The lowest prevalence was found in Greece where 1% of parents admitted to use antibiotics without prescription for their children [19]. On the other hand, the highest prevalence of ASM was found in a Saudi article where Al-Ayed M. S. Z. et al. found a prevalence of parents purchasing antibiotics without prescription of 69% [20] (Figure 2).

**Figure 3:**
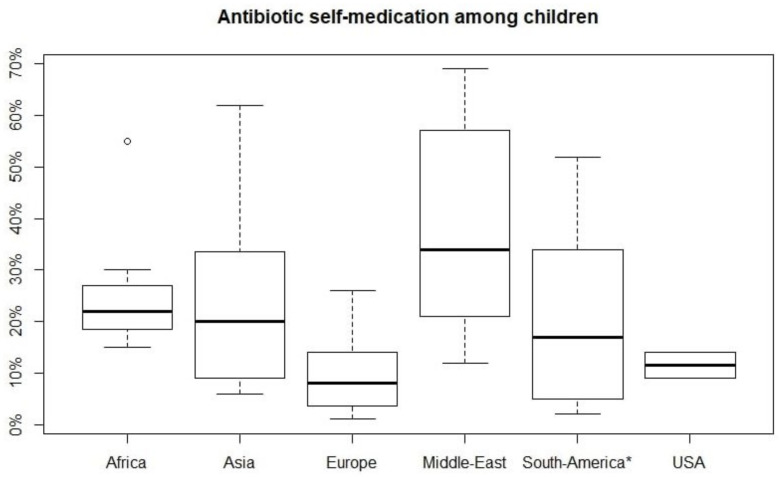
Distribution of prevalence of antibiotics self-medication represented by box plot in world regions. Median (middle black line), confidence region (box), and the maximum non-outlying envelope (whiskers). The circles are the outliers. * Caribbean study was included in South America.

A wide range of prevalence of antibiotics self-medication was found, but generally it was higher in some countries than in others. A box plot was created to analyze the distribution of ASM in the world region from which the studies were conducted (Figure 2). A higher prevalence was found in Middle East (34%), Africa (22%), Asia (20%), and South America (17%), while the lowest prevalence was found in Europe (8%) [18,19,20,21,22,23,24,25,26,27,28,29,30,31,32,33,34,35,36,37,38,39,40,41,42,43,44,45,46,47,48,49,50,51,52,53,54,55,56,57,58,59,60,61,62,63,64,65,66,67,68,69,70,71,72] (Figure 3).

### 2.3. Parents’ Characteristics and Association with Antibiotic Self-Medication

Many studies highlighted that none or few of the parent’s features are significantly related with antibiotics self-medication. Nevertheless, some articles found that significant association between parents characteristics and ASM.

Parents’ main characteristics such as age, living in a rural area, socioeconomic status, relationship with child, level of instruction and occupation, were often obtained.

Mothers answered 68% of questionnaires or interviews, on average. Generally, age, socioeconomic status, and level of instruction were obtained using categorical variable, while questions about occupation were focused on distinguishing who worked in a medical field.

#### 2.3.1. Parents Relationship with Children

Two studies discovered that the risk of ASM was significantly higher for mothers than fathers [40,46], but other three studies found that the risk of self-medication was higher in fathers than in mothers [47,57,63].

#### 2.3.2. Age of Parents

Only two study found that parent’s age resulted significantly related to self-medication. In the first one, from Tanzania [56], researchers found that parents younger than 40 tended to administer more antibiotics without consulting a physician. On the contrary, in a study from Jordan parents older than 40 were more inclined to self-medicate their children with antibiotics [45].

#### 2.3.3. Socioeconomic Status

Low and middle income were statistically related with a high risk of antibiotics self-medication [21,45,46,65,66] with a maximum odds ratio of 4.44 (1.52 to 18.95) [66], while high economic status reduced the risk of self-medication [57,61] such as having an health insurance [65]. In addiction, Palmer D.A. et al. demonstrated that self-medication risk increased if parents attended a Community Health Center instead of a private center [70]. Conversely, two studies demonstrated that medical insurance increased the risk of antibiotic self-medication in children with an odds ratio of 2.31 and a confidence interval (C.I.) of 1.38–4.02 and of 1.30 (C.I. 1.05–1.61), respectively [27,29].

#### 2.3.4. Educational Level

There are many studies which considered a medium or high level of instruction as a protective factor. For example both in a Brazilian study and in a Chinese study, high-school degree significantly reduced the risk of ASM [57,64]. The same result was observed in a Jordan study were parents who have attended university had a reduced risk of antibiotic self-medication [47]. Moreover, a high level of instruction turned out to be a risk factor for ASM only for mothers in a Chinese study [27] and in a German study (OR 1.37, IC 1.19–1.57) that considered both children and adolescents [69].

#### 2.3.5. Working in a Medical Field

Despite Mukattash, T. L. et al. demonstrated that working in a medical field resulted to be a protective factor against AMS with an odds ratio (OR) < 1 (*p* < 0.001) [45], two Chinese studies found that this factor was associated with antibiotics self-medication with an odds ratio of 2.74 (1.080–7.077) and an odds ratio of 1.38 (1.14–1.66), respectively [29,65].

#### 2.3.6. Accessibility to Health Services

Distance from hospital and living in a rural area was associated with antibiotic self-medication. In an Ugandan article the risk of self-medication was 3.7 times higher (C.I. 1.86–7.22) for those parents who lived in rural areas [47]. In a Tanzanian article, a distance >30 Km was statistically associated with ASM (OR 1.2; C.I. 1.1–1.3) and a similar result was found in an American and in a Chinese study, respectively [34,56,62].

### 2.4. Parents’ Knowledge Associated with Antibiotics Self-Medication

#### 2.4.1. Antimicrobial Resistance

On average, 53% of parents, ranging from 11.3% [33] to 90% [71], knew the problem of antimicrobial resistance [20,25,36,37,39,42,45,48,51,53,54,55,58,59,62,67,70,72].

#### 2.4.2. Symptoms Affecting ASM in Children

From 6.4% [35] to 85% [39] of parents thought that antibiotics were indicated to relief symptoms such as fever, cough, runny nose, abdominal pain, or common cold [20,25,32,33,35,37,39,42,48,53,59,62,66,67,70,71,72] and this was associated with antibiotic self-medication in a Saudi study with an odds ratio of 2.17 (C.I. 1.19–3.96) [21].

#### 2.4.3. Antibiotics to Treat Viruses

Over 60%, ranging from 17.9% [35] to 92% [32], of parents believed that antibiotics were useful to treat disease typically provoked by viruses [20,25,32,35,37,38,39,42,45,48,51,53,54,55,58,59,62,67,70,71,72]. In a Caribbean study, it was demonstrated that parents with low knowledge about antibiotics (Caregivers’ Antibiotic Knowledge Score (AKS)<12) had an higher risk to self-medicate their children [51].

#### 2.4.4. Ability to Recognize Antibiotics

From 32.5% [20] to 80% [33] of parents recognized common antibiotics such as amoxicillin [20,33,42,66,72]. The ability to recognize antibiotics was also related with ASM by Lin L. et al. in a Chinese study where parents with a medium or high ability to recognize antibiotics tended to self-medicate their children: OR 1.55 (C.I. 1.14–2.11), OR 1.73 (C.I. 1.31–2.29), respectively [41].

### 2.5. Parents’ Attitude Associated with Antibiotics Self-Medication

#### 2.5.1. Management of Leftover Antibiotics

On average, about 40%, ranging from 12% [47] to 80.5% [65], of parents interviewed believed that leftover antibiotics used to treat their children or an other member of their family in the past could be used to treat their children [21,37,45,47,50,51,53,54,55,61,62,66,72]. This belief was statistically associated with higher risk of ASM with an odds ratio of 3.01 (C.I. 1.77–5.37) [21].

#### 2.5.2. Experience with Antibiotics

Some studies demonstrated that parents who had some previous experience with antibiotics tended to self-medicate their children with those drugs [18,22,55]. In a Mongolian study, mothers who had already medicated children with antibiotics, had an increased risk of ASM with an odds ratio of 6.3 (C.I. 3.8–10.5) [59]. Moreover it was demonstrated that being used to self-medication was statistically associated to an increased risk of antibiotic self-medication of children [36] as well as using an high number of antibiotics in the last year [37].

#### 2.5.3. Relationship with Physician

Requesting more antibiotics to physician augmented the risk of ASM with an odds ratio of 3.22 (C.I. 1.20–8.63) [60]. In addiction, low confidence with physicians increased the risk of antibiotic self-medication [58].

Finally, Yu M. et al. found that parents who did not know that antibiotics are not over-the-counter drugs, but must be prescribed by a physician, tended to self-medicate their children [62]. A similar observation was found by Chang, J. et al. who highlighted that knowing prescription-only regulation for sales of antibiotics at community pharmacies was a protective factor against ASM in children with an OR of 0.77 (0.66–0.91) [29].

### 2.6. Children Features Associated with Antibiotic Self-Medication

Children characteristics were often considered as possible factors affecting self-medication. Some of the studies considered infant and preschool (0–5 years) [22,23,29,33,39,46,47,56,59,66] or scholar-aged children (6–12 years) [31,49] while all other surveys did not specify the age of the children or included parents whose children were under eighteen [18,19,20,21,24,25,26,27,28,30,32,34,35,36,37,38,40,41,42,43,44,45,48,50,51,52,53,54,55,57,58,60,61,62,63,64,65,66,67,68,69,70,71,72,73,74].

A German survey considering behaviors of children from 0 to 17 was also included [69].

#### 2.6.1. Age of Children

Age of children was associated with antibiotic self-medication in a Peruvian study (OR 1.3, C.I. 1.1–1.4) and in a Chinese study where the OR related to age of child was 1.15, CI 1.04–1.27 [33,62]. Moreover, Yuan, J. et al. found a similar relationship between age of children and ASM for two age ranges: children whose age was between 3 and 5 years had an odds ratio of 1.82 (C.I. 1.15–3.02), while for those was age was between 6 and 11 odds ratio augmented to 2.19 (C.I. 1.40–3.60) [63].

#### 2.6.2. Number of Children

Having more children was a factor that augmented the risk of antibiotics self-medication in several studies. A Chinese and a Jordan study found a significant relationship with having more than one child and antibiotic self-medication; the Chinese study reported an odds ratio of 2.17 (C.I. 1.48–3.18) [47,62]. Moreover, a Saudi study found a greater risk of antibiotic self-medication in those parents with more than two children with an odds ratio of 1.68 (C.I. 0.99–2.85) [21].

#### 2.6.3. Children Symptoms

**Figure 4:**
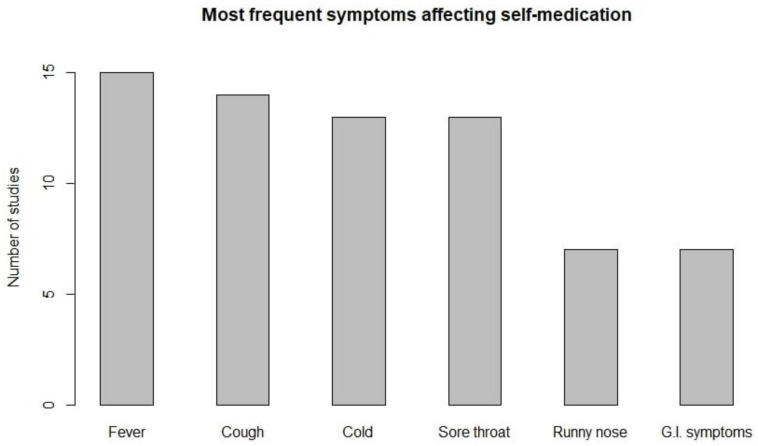
Frequency of symptoms affecting self-medication among parents: Fever (n = 15), cough (n = 14), cold (n = 13), sore throat (n = 13), runny nose (n = 7), and G.I. symptoms (n = 7).

Other child factors taken into account and influencing antibiotics self-medication were. Fever, cough, common cold, and sore throat were the most frequent symptoms, followed by runny nose and gastrointestinal symptoms (Figure 4).

On average, about an half of parents who practiced ASM administered an antibiotic to relief common cold or cough, while this percentage reduced to 40% for fever and sore throat [18,19,22,26,27,30,31,34,40,41,42,43,46,47,50,51,54,55,56,58,59,60,61,64,69,70,71,74].

Moreover, a study highlighted that there were symptoms causing a greater risk of self-medication. Fever appeared to be a risk factor for self-medication in a Chinese study with an odds ratio of 1.89 (C.I. 1.58–2.26) [41]. Cough was statistically related to ASM in an Ugandan study where the odds ration was 3.54 (C.I. 1.55–8.06) [47]. In this study, diarrhea was also a condition affecting self-medication, with an odds ratio of 8.00 (C.I. 3.31–19.30). Moreover, Zhu, Y. et al. found that runny nose turned out to be a possible risk factor [66].

#### 2.6.4. Children Health Status Perception

Pfaffenbach G. et al. associated the practice of self-medication with personal health state perception. In particular, despite the fact that this study also focused on adolescents, it was demonstrated that the children or adolescents who considered their health status low tended to practice more self-medication [69]. A similar result was found in two Chinese studies: the first one highlighted that parents who considered their children’s health status “good” or “very good” had low risk of ASM (OR 0.48, C.I. 0.40–0.57) [29], while the second one found that medium/high severity of children auto-diagnosed disease OR 1.76 (C.I. 1.40–2.23) [41].

### 2.7. Source of Information

Many studies have investigated parents sources of information about the correct use of antibiotics.

#### 2.7.1. Physicians

The main source of information were physicians. In fact, in three European studies [71,72,73] the percentage of parents whose main source of information was physician ranged between 80% and 90%. This result was similar in a Chinese study where physicians were the main source of information for 70% of the sample [62], while in a Pakistani study this percentage was around 90% [26]. By the way, sometimes physicians were not the main source of information. For example, in a Nigerian study, just one third of parents obtained information about the correct use of antibiotics from doctors [50] and in a Pakistani study, family, friends, and pharmacists were a greater source of information about the correct use of antibiotics [41].

#### 2.7.2. Pharmacists

The second most important source of information was a pharmacist. Especially, in some studies, a great percentage of parents search for information in a pharmacy [40,58]. In a European study, the amount of parents who received information about the use of antibiotics by a pharmacist was 15%. The same result was seen in an Indian study. On the contrary many articles reported percentage of information by pharmacist from 24% to 63% [25,35,43,62,65,73]. In addiction, in a Peruvian study, the odds ratio of self-medication was 3.0 for parents who received information in pharmacy with a significant confidence interval (1.9–4.6) [22].

#### 2.7.3. Other Source of Information

Other source of information reported were: television and mass media [43,72], drug leaflet [37,73], family and friends [43,50,65], and the advice from a third-party was significantly related to antibiotics self-medication in a Cameroonian study (*p* < 0.05) [35]. Finally, a Saudi article highlighted that around 33% of parents search for information on the Internet, which was an important source of information in a Greek study too (37%) [25,72].

### 2.8. Source of Antibiotics

Most of these studies reporting information regarding the source of antibiotics described two main ways by which parents obtained antibiotics, which are discussed as follows.

#### 2.8.1. Purchasing Antibiotics without Prescription

This was a worldwide practice found in various articles from Africa, Asia, and the Middle East, but also in Europe (Macedonia) [18,20,36,38,45,47,55,56,66,68,74] and it was significantly related to the practice of self-medication in a Chinese study with an odds ratio of 1.15 (C.I. 1.01–1.30) [27].

#### 2.8.2. Leftover Antibiotics from Previous Prescribed Treatment

This practice was found less frequently and, in general, it was a marginal source of antibiotics: 4% in a Macedonian study, 12% in an Egyptian study, 14.7% in a Emirate study, and 15.7% in a Chinese study [36,38,54,66]. There was also an exception represented by an Indian article where the percentage of parents who used leftover antibiotics for their children self-medication was over one third [68].

Zhu Y. et al. [66] described an other practice in Yiwu, a city of about 2 million people situated in the central Zhejiang Province of China: about one third of parents who self-medicated their children tend to stock antibiotics at home.

## 3. Materials and Methods

### 3.1. Search Strategy and Selection Criteria

The systematic review was conducted following the PRISMA checklist by searching PubMed, Scopus, and Web of Science until July 2022, no limit was set as to the year of publication or study location. It was registered with the Open Science Framework (OSF).

The review was conducted according to the PRISMA guidelines that ensure transparency, accuracy, and complete reporting of systematic reviews [75]. Two researcher conducted the initial screening of title and abstract, evaluated independently all the screened full-text article and finally extracted the data to conduct semi-quantitative analysis and quality analysis of studies.

Three databases were searched: PubMed, Scopus, Web Of Science. Search terms included both MeSH terms and free text (keywords, synonyms, and word variations), connected with Boolean operators. Specifically, we applied “OR” in each group of keywords and MeSH terms to identify the areas of interest and “AND” operator to combine each group. Strings used for each database are available as Supplementary Materials.

### 3.2. Eligibility Criteria

All types of studies designs were included if they met the following inclusion criteria:

- Study was available in English language.
- Study contained information about parental attitude to antibiotics self-medication.
- Study focused on infant, pre-school, or scholar-age children.

Studies that dealt with antibiotics self-medication in both children and adolescents were also included.

### 3.3. Definitions

According to National Library of Medicine:

- Self-medication consists in the self administration of medication not prescribed by a physician or in a manner not directed by a physician.
- Antibiotics or anti-bacterial agents are defined as substances that inhibit the growth or reproduction of bacteria.
- Children are divided by age in: infant (less than 2), pre-school (between 2 and 5), and school-age (between 6 and 12).

### 3.4. Data Extraction and Management

The screening of search results was performed using the web-based, open-access platform Rayyan (https://www.rayyan.ai/, accessed on 25 July 2022). Data were independently extracted by two authors into pre-defined and labeled columns in an Excel spreadsheet. Data extracted include the nation, population, socio-demographic and socio-economic characteristics of the parents, and their knowledge, attitudes, and behaviors related to self-administering antibiotics to their children. Proportion of attitude or practice of antibiotic self-medication was always obtained. Bar chart, box plot, and map were obtained processing data on R software [76].

## 4. Discussion

The phenomenon of antimicrobial resistance is a rapidly progressing problem worldwide. In recent years, major public health agencies have highlighted the urgency of monitoring and investigating this phenomenon to counter the spread of antibiotic-resistant microorganisms [1,2,3,4,5]. The misuse of these drugs, as well as self-medication, are the main causes of this phenomenon. In particular, self-medication in children, precisely because of their greater susceptibility to airway infections, can play a key role in combating the spread of AMR. Understanding the socio-cultural, geographical, and economic variables that may influence parents’ behavior regarding self-medication of their children is essential for planning future health education interventions aimed at contain the overuse and misuse of these drugs.

In this systematic review, which includes studies from all over the world, significant differences regarding ASM emerged. In particular, it was observed that in some geographic areas, the practice of antibiotic self-medication in children is frequent. One of the causes that may partly justify these differences on a macro-regional level is the different possibility of purchasing antibiotics without a prescription [77,78,79,80].

The regions showing high rates of antibiotics bought without a prescription seem to overlap geographically with the same ones that showed a higher prevalence of antibiotic self-medication in children. In recent years, many countries have introduced stricter regulations on the sale of antibiotics, but the prevalence of drugs sold without a prescription remains high. This is mainly due to non-compliance by community pharmacies with existing laws. Despite the severe punishments provided, these interventions do not seem to be decisive [78]. Therefore, in these contexts, community pharmacists trained in antibiotic stewardship could play a significant role in ensuring rational use of antibiotics [80].

In addition to the legislative aspect, the common cultural denominator, at the level of the general population and public health policies, may play a key role in explaining this phenomenon. In particular, this can be partially appreciated by comparing the sales of antibiotics without prescription in some macro-regions with the prevalence of self-medication in children. For example, in some European countries, it is possible to buy antibiotics without a prescription, although it is not legal, but the prevalence of ASM in children is low (Figure 3).

This is probably related to a higher average level of education and more developed health and welfare policies. As shown in our study, a high level of parental education is a potential protective factor. Children of these parents have a lower risk of receiving nonprescription antibiotics (Table 1). In addition, another aspect that influences attitudes toward antibiotic self-medication in children is the accessibility of healthcare. In particular, physical distance from health care services in rural areas may cause under-utilization of these services and encourage self-medication behaviors such as storing previously used drugs. Furthermore, considering the non-universality of most health care systems, parents’ difficulty in bearing the cost of medical consultation may be a risk factor for self-medication of antibiotics in children (Table 1). To overcome these difficulties, many parents reported asking pharmacists for information about antibiotic use.

Having more children could increase the risk of self-medication (Table 2). Rather than an independent risk factor, this may be the result of multiple factors that are enhanced by having more than one child. As just mentioned, access to facilities is not always guaranteed, whether due to physical distance or economic status. This is more important in case parents have more than one child. In addition, since previous experience in antibiotic use is a risk factor for ASM (Table 1), having more children can promote this behavior. This is because those parents with two or more children are more likely to have administered an antibiotic in the past than those with only one child. The probability of having leftover antibiotics in pediatric formulation at home is higher too.

The age of the children seems to influence parental behavior, as an increase in age would increase the risk of self-medication. This could be linked to a lower perception of the risk of side effects in scholar-age children (Table 2). On the contrary, parental age appears not to be a risk factor for ASM as only two studies showed statistical significance, but the results were diametrically opposed.

Considering the symptoms that most frequently promote self-medication in children, such as fever, cough, and sore throat, these symptoms are often caused by viruses and do not represent an indication for the use of antibiotic therapy. Nevertheless, they are among the symptoms that most frequently elicit parents to practice ASM in children (Table 2). As mentioned earlier, parental education plays a key role in this context. In fact, it was seen that among parents who had appropriate knowledge about the use of antibiotics, the prevalence of self-medication was lower. Although there are limited data, this does not appear to be true for health-care workers. In fact, in this category there is a higher prevalence of parents administering antibiotics without a prescription to their children (Table 1). On the other hand, a lower comprehension of the risks associated with self-medication remained among parents who were able to recognize antibiotics or had previous experience handling antibiotics (Table 1). This highlights that, among the measures to combat self-medication in children, in particular and anti-microbial resistance in general, it is important to involve parents through education and training programs. In fact, is fundamental to note that parents’ knowledge about the proper use of antibiotics was found to be generally low. For example, only 50% of parents on average were unaware of the problem of AMR. Good practices on the correct use of antibiotics could be spread through television and the mass media that many parents reported to be important source of information. Conversely, the acquisition of information through family and friends should be discouraged as it has been correlated with an increase in bad practice in the use of antibiotics.

The main limitation of this review is the poor representation of some macro-regions or continents. We cannot exclude the possibility that studies were conducted mainly where the problem of inappropriate antibiotic use is most felt (publication bias). In addition, the extracted data are mainly derived from surveys. Moreover, there are no studies correlating the type of antibiotic or pharmaceutical form with self-medication practice in children. For these reasons, further studies are needed to better understand the extent of the problem globally.

In conclusion, although antibiotic self-medication among children is a global phenomenon, influenced by a number of geographical, cultural, and economic factors, there is an urgent need to promote a worldwide health strategy.

Specifically, greater attention to the regulation of the sale of antimicrobial drugs can partially limit the risk of self-medicating behavior. The introduction of health education programs, specifically aimed at parents and pharmacists, can at the same time, improve understanding of the risks associated with self-medication.

Lastly, constant monitoring of these phenomena, and raising stakeholders’ awareness of the practices that lead to antibiotic resistance, may favor a more careful use of these drugs in the near future.

## Figures and Tables

**Table 1:** Parents characteristics, knowledge, and attitude associated with self-medication.

Features Associated with ASM	Risk of ASM ^1^	Citation
Child relationship: Mother	OR 0.30 (0.09–0.96)	Nyeko et al. [47]
	OR 0.83 (0.74-0.94)	Sun et al. [57]
Child relationship: Father	OR 0.74 (0.4–1.3)	Abdulaziz H et al. [21]
	OR 0.53 (0.3–0.96)	Zhu et al. [66]
	OR 1.27 (1.1–1.5)	Yuan et al. [63]
High distance from hospital/Rural Area	OR 3.70 (1.9–7.2)	Nyeko et al. [47]
	OR 1.20 (1.1–1.3)	Simon and Kazaura [56]
	OR 1.60 (1.1–2.4)	Yu et al. [62]
Low/middle Economic Status	OR 4.44 (1.5–19.0)	Zhu et al. [66]
	OR 3.60 (1.3–9.7)	Zhou et al. [65]
	OR 2.00 (1.1–3.8)	Abdulaziz H et al. [21]
High Economic Status	OR 0.66 (0.5–1)	Sun et al. [57]
Having medical insurance	OR 0.36 (0.1–1)	Zhou et al. [65]
	OR 2.31 (1.4–4.0)	Bi et al. [27]
	OR 1.30 (1.1–1.6)	Chang et al. [29]
High level of instruction	OR 0.71 (0.5–1)	Sun et al. [57]
	OR 0.34 (0.2–0.5)	Zhang et al. [64]
Working in medical field	OR 1.38 (1.1–1.7)	Chang et al. [29]
	OR 2.74 (1.1–7.1)	Zhou et al. [65]
Medium ability to recognize antibiotics	OR 1.55 (1.1–2.1)	Lin et al. [41]
High ability to recognize antibiotics	OR 1.73 (1.3–2.3)	Lin et al. [41]
Tendency toward self-medication	OR 6.30 (3.8–10.5)	Togoobaatar et al. [59]
Requesting antibiotics to physician	OR 3.22 (1.2-8.6)	Xu et al. [60]
Knowing antibiotics should be prescribed	OR 0.77 (0.7–0.9)	Chang et al. [29]

^1^ Only significant odds ratios, with their confidence interval were inserted in the table.

**Table 2:** Children’s characteristics associated with self-medication.

Features Associated with ASM	Risk of ASM ^1^	Citation
Age of children	OR 1.30 (1.1–1.4)	Ecker et al. [33]
	OR 1.15 (1.1–1.3)	Yu et al. [62]
Having more than one child	OR 2.17 (1.5–3.2)	Yu et al. [62]
	OR 1.68 (1.0–2.9)	Abdulaziz H et al. [21]
Children poor health status	OR 2.10 (1.75–2.5) ^2^	Chang et al. [29]
	OR 1.76 (1.40–2.23)	Lin et al. [41]
Fever	OR 1.89 (1.6–2.3)	Lin et al. [41]
Cough	OR 3.54 (1.6–8.1)	Nyeko et al. [47]
Diarrhea	OR 8.00 (3.3–19.3)	Nyeko et al. [47]
Runny Nose	OR 1.86 (1.13–3.19)	Zhu et al. [66]

^1^ Only significant odds ratios (OR), with their confidence interval (CI) were inserted in the table; ^2^ The reciprocal odds ratio was calculated.

## Data Availability

Data is contained within the article or Supplementary Material.

## Supplementary Materials

The following supporting information can be downloaded at: https://www.mdpi.com/article/10.3390/antibiotics11111583/s1, search strings.

## Abbreviations

The following abbreviations are used in this manuscript:

AMR	Anti Microbial Resistance
ASM	Antibiotic Self-Medication
CI	Confidence Interval
OR	Odds Ratio
