# Supplementary material for: Antibiotics Self Medication among Children: A Systematic Review
Source: Antibiotics (Basel). 2022 Nov 9;11(11):1583. doi: 10.3390/antibiotics11111583 (PMC9687075; doi:10.3390/antibiotics11111583)
Supplement: Supplementary file 1 [file antibiotics-11-01583-s001.zip › antibiotics-1968110-supplementary.pdf]

5.1 Pubmed

Free text terms and MESH terms included were the following:
(”anti bacterial agents”[Pharmacological Action] OR ”anti bacterial agents”[MeSH Terms] OR (”anti bacterial”[All Fields] AND ”agents”[All Fields]) OR ”anti bacterial agents”[All Fields] OR ”antibiotic”[All Fields] OR ”antibiotics”[All Fields] OR ”antibiotic s”[All Fields] OR ”antibiotical”[All Fields]) AND (”self medication”[MeSH Terms] OR (”self”[All Fields] AND ”medication”[All Fields]) OR ”self medication”[All Fields]) AND (”child*”[All Fields] OR (”parent s”[All Fields] OR ”parentally”[All Fields] OR ”parentals”[All Fields] OR ”parented”[All Fields] OR ”parenting”[MeSH Terms] OR ”parenting”[All Fields] OR ”parents”[MeSH Terms] OR ”parents”[All Fields] OR ”parent”[All Fields] OR ”parental”[All Fields])).

5.2 Web of Science

Free text terms and MESH terms included were the following:
antibiotics AND (self medication) AND (child* OR preschool)

5.3 Scopus

Free text terms and MESH terms included were the following:
TITLE-ABS-KEY ((self-medication OR self-prescription) AND antibiotic* AND (parent* OR child* OR preschool))
